# The intrinsically disordered Tarp protein from chlamydia binds actin with a partially preformed helix

**DOI:** 10.1038/s41598-018-20290-8

**Published:** 2018-01-31

**Authors:** James Tolchard, Samuel J. Walpole, Andrew J. Miles, Robin Maytum, Lawrence A. Eaglen, Ted Hackstadt, B. A. Wallace, Tharin M. A. Blumenschein

**Affiliations:** 1 Center for Molecular and Structural Biology, School of Chemistry, University of East Anglia, Norwich Research Park, Norwich, NR4 7TJ UK; 2 Institute of Structural and Molecular Biology, Birkbeck College, University of London, London, WC1E 7HX UK; 3 School of Life Sciences, University of Bedfordshire, Park Square, Luton, LU1 3JU UK; 4 Host-parasite Interactions Section, Laboratory of Intracellular Parasites, NIAID, NIH, Rocky Mountain Laboratories, Hamilton, MT 59840 USA

## Abstract

Tarp (translocated actin recruiting phosphoprotein) is an effector protein common to all chlamydial species that functions to remodel the host-actin cytoskeleton during the initial stage of infection. In *C. trachomatis*, direct binding to actin monomers has been broadly mapped to a 100-residue region (726–825) which is predicted to be predominantly disordered, with the exception of a ~10-residue α-helical patch homologous to other WH2 actin-binding motifs. Biophysical investigations demonstrate that a Tarp_726–825_ construct behaves as a typical intrinsically disordered protein; within it, NMR relaxation measurements and chemical shift analysis identify the ten residue WH2-homologous region to exhibit partial α-helix formation. Isothermal titration calorimetry experiments on the same construct in the presence of monomeric G-actin show a well defined binding event with a 1:1 stoichiometry and K_d_ of 102 nM, whilst synchrotron radiation circular dichroism spectroscopy suggests the binding is concomitant with an increase in helical secondary structure. Furthermore, NMR experiments in the presence of G-actin indicate this interaction affects the proposed WH2-like α-helical region, supporting results from *in silico* docking calculations which suggest that, when folded, this α-helix binds within the actin hydrophobic cleft as seen for other actin-associated proteins.

## Introduction

Chlamydiae are obligate intracellular pathogens which cause a significant disease burden in humans. *Chlamydia trachomatis* is currently the most prevalent bacterial sexually transmitted disease worldwide^1,2^, and is also responsible for trachoma, the leading cause of preventable blindness in the developing world^3^. The mechanism by which chlamydiae gain entry to host cells involves a multi-domain, type-III secreted protein termed Tarp (translocated actin-recruiting phosphoprotein) that remodels the host cell actin cytoskeleton, causing a phagocytosis-like internalization of the infectious chlamydial particles (elementary bodies, EB)^4^. All chlamydial species contain orthologues of the Tarp protein and, although variation is observed in domain multiplicity, most variants characterized to date contain distinct F and G-actin-binding domains^5^, a proline-rich oligomerization domain and a tyrosine rich phosphorylation domain^6,7^. The basic unit of the tyrosine-rich repeat contains 50 amino acids but the number of repeats varies in different species and across *C. trachomatis* serovars^8^. Interestingly, this region is missing completely in *C. muridarum*, *C. caviae* and *C. pneumoniae*, with no apparent detriment to infection^9^. The proline-rich domain enables Tarp to form homo-multimers that can facilitate actin filament nucleation at the site of chlamydial invasion by colocalising multiple G-actin binding domains; this is particularly important in the case of *C. pneumoniae* and the *C. trachomatis* L2 serovar whose Tarp proteins only contain one actin binding domain. The importance of colocalisation is further supported by evidence suggesting that Tarp orthologues containing more than one G-actin binding domain possess faster actin nucleation and do not absolutely require oligomerisation to promote actin nucleation^7^. However, it should also be noted that proline-rich domains are also widely implicated in a wide range of protein-protein interactions and protein complex assemblies^10^.

The proposed G-actin-binding region of Tarp, experimentally mapped to within the Tarp_726–825_ construct, is approximately 12 residues in length and shows striking sequence homology to other known actin-binding helices in WH2 (WASP homology-2) domains. Proteins containing the ~17–20- residue WH2 actin binding domain are usually involved in the regulation of cytoskeletal dynamics and occur across a wide range of eukarya with the exception of plant species^11^. Canonical WH2 domains contain a short N-terminal amphiphilic α-helix, followed by an extended region containing the motif LKKT^12^. Existing structures of actin-bound WH2 domains typically present the helical region binding to the actin hydrophobic cleft between subdomains 1 and 3 whilst the remaining extended residues interact through hydrophobic interactions and salt bridges across the surface of actin subdomain 1, towards subdomains 2 and 4. In Tarp, the homology in the G-actin binding region is restricted to the initial helix. However, the G-actin binding domain is still proposed to interact with actin in a similar fashion to other WH2 domain-containing proteins. It was an objective of this work to investigate which residues are involved in the binding.

Full length Tarp has been shown to directly nucleate actin filaments *in vitro*^13^, supporting its natural function; *C. trachomatis* Tarp is also able to interact with host cell actin-regulating proteins and further contribute to actin polymerization by subverting natural signaling pathways. Tarp’s tyrosine-rich phosphorylation domain has been shown to interact in a phosphate-dependent manner with Rac guanine exchange factors Sos1 and Vav2, involved in the signaling cascade responsible for remodeling of the actin cytoskeleton, leading to the eventual activation of cortactin and the Arp2/3 complex^14,15^. Recent Tarp studies have also identified conserved binding sites for focal-adhesion kinase (FAK)^16^ and the actin-associated protein vinculin^17^, both of which enhance actin recruitment by Tarp, albeit independently. Thus, it has become clear that the Tarp protein is involved in a multitude of protein:protein interactions, to achieve the principal objective of polymerising and bundling actin filaments *en masse* to facilitate host-cell internalization by parasite-specified phagocytosis. Indeed, perturbation of these binding interactions, and those of the phosphorylation domain, have been linked to a reduction in chlamydial pathogenicity in a laboratory environment^7,14,16,17^, and Tarp antibody recognition has been shown to confer protective immunity^18^. A comprehensive review of the known elements of chlamydial pathogenesis, as well as the broad-range of interactions by Tarp has recently been presented by Elwell *et al*.^19^.

Although such results are beginning to reveal the genetic and functional basis for chlamydial pathogenicity, structural biology studies of Tarp and its interactions are still lacking. This work therefore aimed to elucidate the structural and dynamic behavior of the native state of the Tarp G-actin-binding region, and its interaction with G-actin, by the application of solution state nuclear magnetic resonance (NMR) and synchrotron radiation circular dichroism (SRCD) spectroscopies and isothermal calorimetry (ITC). Based on these results, we also propose a definition of the Tarp:G-actin interaction through the construction of a guided computational model.

## Results

### The Tarp G-actin binding domain is intrinsically disordered

**Figure 1:**
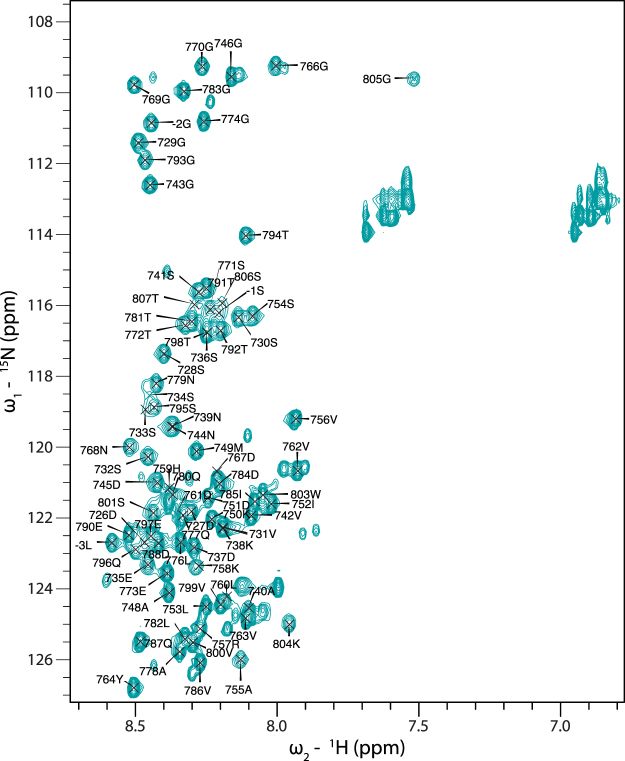
[^1^H-^15^N]-HSQC spectrum of Tarp_726–825_. The W803 side chain amide peak has been cropped out for clarity. Glutamine and asparagine side chain resonances (^1^H: 6.8–7.7 ppm) were not assigned. Assignments refer to wild-type, full-length Tarp residue numbering and negative numbers indicate remnants of the GST tag cleavage site. The spectrum was acquired at 800 MHz.

The 2D [^1^H,^15^N]-HSQC spectrum of ^15^N-labelled Tarp_726–825_, a construct containing the G-actin binding domain (Fig. 1), shows extremely narrow spectral dispersion in the ^1^H dimension of the backbone amide chemical shifts (majority of peaks within ~0.7 ppm). This is a typical characteristic of an intrinsically disordered protein (IDP)^20^. In further support of this disordered nature, the 3D [^1^H,^15^N]-HSQC-NOESY spectrum also lacks non-sequential correlations (Supplementary Fig. S1). In addition, ourselves and others have observed Tarp to migrate aberrantly by SDS-PAGE^6^ and size exclusion chromatography (Supplementary Fig. S2), another common characteristic of IDPs.

Using triple resonance NMR experiments, backbone atoms for 82% of the Tarp_726–825_ residues could be specifically assigned. The C-terminal 18 residues could not be observed. To exclude the possibilities of truncated translation, incorrect purification, or degradation, a sample of the 100-residue construct was analyzed by mass spectrometry and confirmed to be the vastly predominant species present (data not shown), suggesting that the residues could not be observed due to conformational exchange in a timescale unfavourable to direct observation by NMR. The presence of weak peaks for the last residues observed, and of additional weak, unassignable peaks in the [^1^H,^15^N]-HSQC spectrum, reinforces this hypothesis.

In an attempt to observe peaks for the remaining residues, we acquired [^1^H,^15^N]-HSQC spectra at temperatures ranging from 278 to 308 K, with no new peaks appearing (Supplementary Fig. S3). Variations in pH, from 4.5 to 9.9, did not result in any new peaks potentially corresponding to residues between 808 and 825 either (data not shown).

The SRCD spectrum of Tarp_726–825_ (Supplementary Fig. S4) is also consistent with a secondary structure that is primarily instrinsically disordered. Furthermore, thermal-melt SRCD studies of the construct also show behaviour typical of an IDP: the spectral changes (increase in the 224 nm shoulder and decrease in the peak around 190 nm, Supplementary Fig. S4) correspond to a decreased calculated disordered content with increasing temperature, a behaviour that directly contrasts with that seen for most fully folded (not IDP) proteins.

### Tarp_726–825_ contains a region of helical propensity

Neighbour corrected random coil indexing (RCI)^21^, which uses chemical shift information to predict flexibility within a protein, suggests a region of increased order between residues 746–759 in Tarp_726–825_, which corresponds to a deviation towards more helical Cα secondary chemical shifts. This agrees with the positioning of an ordered region proposed by *in silico* disorder prediction (Supplementary Fig. S5) and aligns to the α-helical regions of other homologous WH2 domains that have been shown to interact with the actin hydrophobic cleft. The average Cα secondary chemical shift for fully helical regions in well-folded proteins is 2.7 ppm^22^ suggesting that the WH2-like helix is at most 25% populated in Tarp_726–825_.

**Figure 2:**
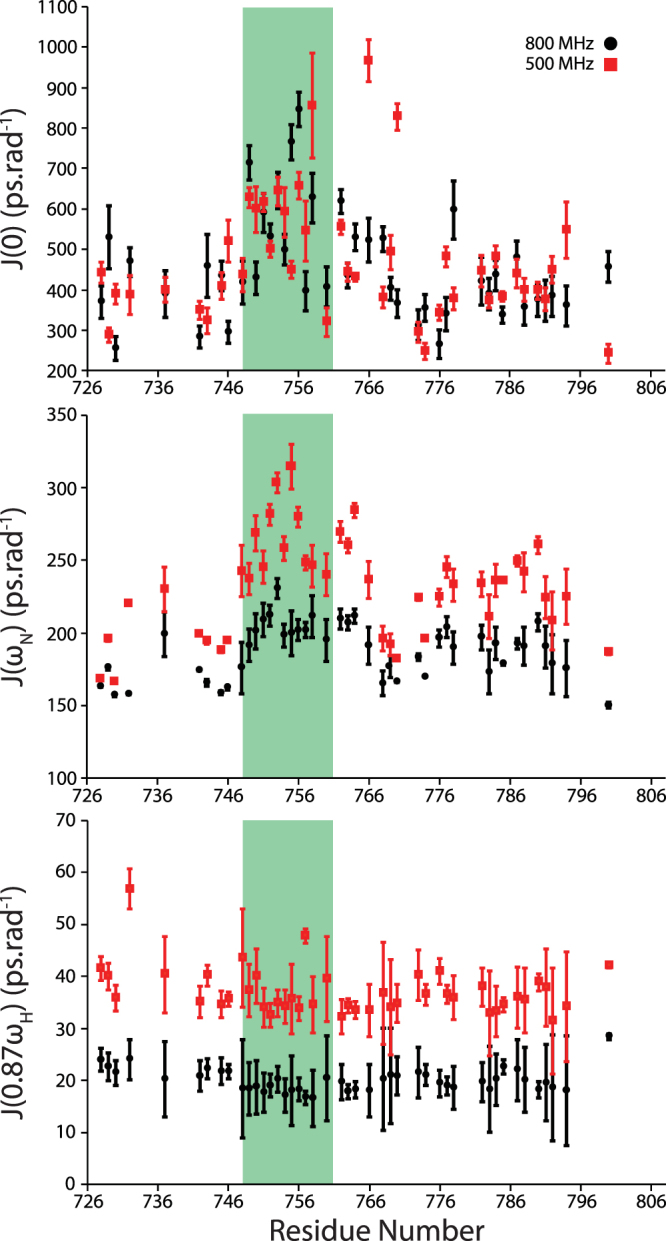
Reduced spectral density analysis of Tarp_726–825_. Spectral density at J(0), J(ω_N_) and J(0.87ω_H_) was calculated from ^15^N relaxation data. Data were acquired to 500 MHz (red squares) and 800 MHz (black circles), and the highlighted green region corresponds to the region of increased helical propensity. Error bars were propagated from the uncertainties in each relaxation measurement.

This partially ordered region was further confirmed by ^15^N relaxation NMR experiments. T_1_, T_2_ and heteronuclear NOE relaxation experiments were acquired at 500 and 800 MHz (Supplementary Fig. S6), and reduced spectral density mapping was employed to extract richer dynamic information. This formalism provides an improved measure of dynamic motion, compared to other dynamic analyses that require *a priori* knowledge, or assumptions, about the nature of motions within the molecule^23^. By extracting the extent of molecular motions at the (field-dependent) frequencies inherent to the NMR observation of ^1^H and ^15^N nuclei, we were then able to probe the accessible molecular dynamics across slow (J(0), ms-μs), intermediate (J(ω_N_), ns) and fast (J(0.87ω_H_), ns-ps) timescales. The calculated reduced spectral density parameters (Fig. 2) suggest that in the fast motional regime, the scope of molecular motions is uniform across the observable Tarp_725–826_ backbone. However, a region with enhanced motion in the slow and intermediate timescales is readily apparent. This region corresponds to the region of helical propensity suggested from *in silico* prediction, the ordered region implied by chemical shift analysis and the stretch of primary sequence homologous to other WH2 binding regions (*vide infra*).

### Tarp binds G-actin with high affinity and increases in helical content

**Figure 3:**
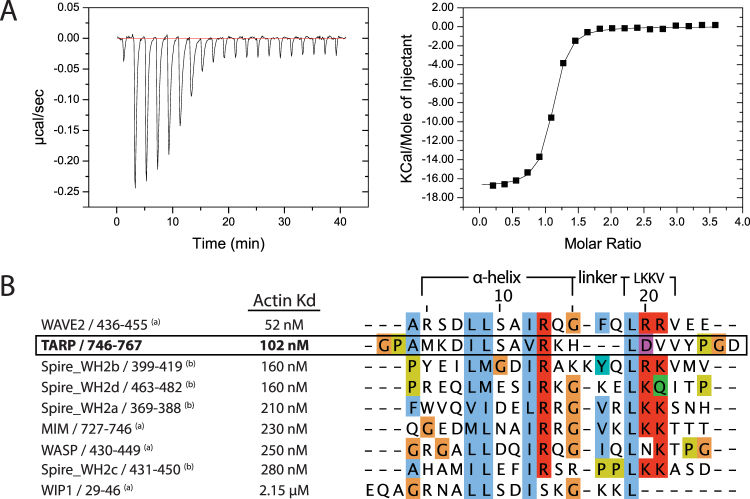
Binding of Tarp_726–825_ to actin relative to other WH2 domains. (**A**) A typical ITC binding isotherm between Tarp_726–825_ and G actin (left) and the integral of the heat change fitted to a single binding site model (right). The enthalpies associated with the dilution of Tarp_726–825_ were subtracted prior to integration. (**B**) Clustal Omega^54^ sequence alignment between the predicted Tarp actin-binding region and other WH2 domains (residue numbers indicated) with published, (a)^12^ or (b)^55^, dissociation constants for binding to actin in a binary complex. Conserved residues are colored by amino acid type, according to the Clustal X scheme.

Tarp_726–825_ has previously been shown to contain the minimal region necessary for interaction with G-actin monomers^13^. To probe the thermodynamics of this interaction, we carried out isothermal titration calorimetry experiments (Fig. 3A) between Tarp_726–825_ and G-actin. The observed isotherm shows an excellent fit to a 1:1 binding event, with a dissociation constant of 102 ± 33 nM, in good agreement with the nanomolar range of other WH2 domain:actin interactions (Fig. 3B and Supplementary Table S1).

The positive –T∆S entropy term (6.56 ± 0.91 kcal mol^−1^) from ITC indicates that a significant decrease in entropy (disorder-to-order transition) is concomitant with binding. For globular protein interactions, this is often a result of reduced molecular mobility, but is also commonly observed for disordered proteins which undergo a folding transition concomitant to binding^20^, and could indicate that the Tarp_726–825_ α-helix becomes more prevalent in the bound state.

**Figure 4:**
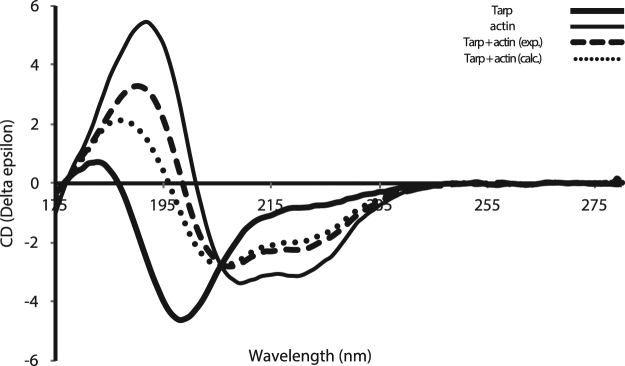
Synchrotron radiation circular dichroism (SRCD) spectra of Tarp_726–825_ binding to actin. Spectra of Tarp_726–825_ (thick line), G-actin (thin line) and a 3.6:1 complex of the Tarp:G-actin (dashed line) are shown. The calculated SRCD Tarp:G-actin spectrum (assuming no change in secondary structure, dotted line) is also shown for comparison with the measured spectrum.

Nonetheless, entropic contributions from actin stabilization and any interactions between actin and the extended region of Tarp_726–825_ cannot be fully discounted. To this end, changes in the α-helical secondary structure content upon binding were also specifically investigated by SRCD spectroscopy. The acquisition of circular dichroism spectra using a synchrotron light source allows CD measurements to be made at lower wavelengths and offers improvements in signal-to-noise^24^. The low wavelength data is especially valuable for characterizing disordered structures, as the characteristic peaks for this type of structure are detectable in the region between 185 and ~200 nm. SRCD was then used to examine the secondary structure content (Table 1, Fig. 4) of Tarp_726–825_, G-actin and a 3.6:1 Tarp:G-actin complex. This ratio was chosen to ensure that G-actin would be saturated with Tarp, avoiding heterogeneity in the population, and also for practical reasons, as it allowed the final samples from ITC experiments to be used. The structural content analysis suggests that overall ~5% of free Tarp_726–825_ is helical in solution (corresponding to around 5 (range of 2–8) residues). The predicted WH2-homologous α-helical region is ~10 amino acids long, which therefore suggests the predicted α-helix region could be roughly 50% helical in solution. This population is larger than that indicated from the secondary structure analysis of NMR chemical shifts alone, but is nonetheless consistent with partial helix formation in the free state. SRCD data therefore strengthens the argument that helical content is present but only partially formed in the free Tarp construct. In comparison, for SRCD analysis of Tarp_726–825_ in the actin-bound state, the overall helical content of the complex is 4% greater than that expected from the linear combination of actin and Tarp secondary structures, assuming no structural change takes place upon binding. This difference could result from conformational changes in the actin partner, of course, but the many examples of actin crystallographic structures, with and without binding partners in the cleft between subdomains 1 and 3, are remarkably invariable^25^, so are less likely to be the source of the differences seen.

**Table 1:** Average secondary structure content of Tarp, G-actin, and the Tarp:actin complex.

Secondary structure	% Secondary structure content				
	Tarp	G-actin	Tarp:actin_calc_	Tarp:actin_exp_	**∆**_**exp-calc**_
α-helix	5 ± 2	28 ± 0	17	21 ± 1	**4**
β-strand	16 ± 0	24 ± 3	29	29 ± 1	**0**
coil	68 ± 2	34 ± 1	39	37 ± 2	**−2**

For the complex, both experimental and calculated (assuming no secondary structure change in a mixture of the two proteins) values are displayed.

**Figure 5:**
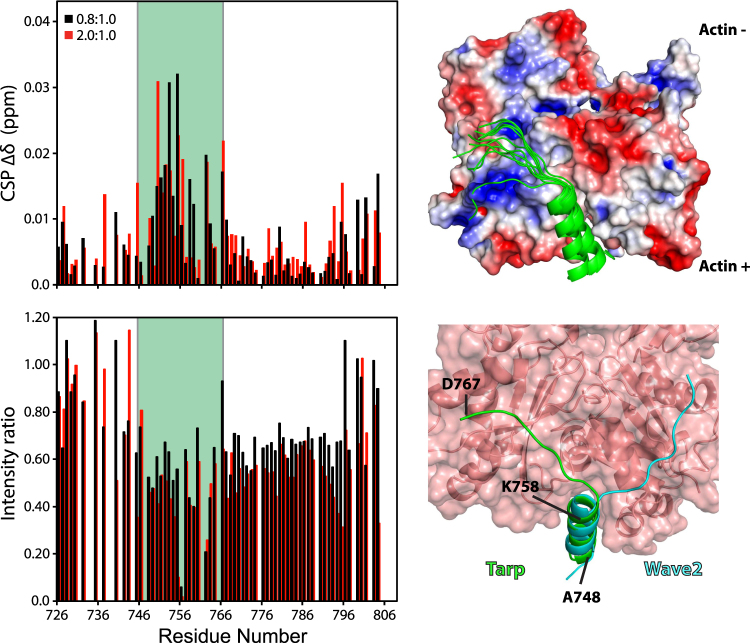
Binding of Tarp to actin. Left: The chemical shift perturbations (∆δ) (top) and relative peak intensity changes (bottom) observed between [^1^H-^15^N]-HSQC spectra of free Tarp_726–825_ and those containing 0.8:1 (black) and 2:1 (red) approximate molar ratios of G-actin. The residues highlighted within the green strip indicate the WH2-homologous Tarp region (residues 746–767) used for the Rosetta based FlexPepDock modeling^26,27^. Top right: The 10 lowest energy complex models of the docked Tarp WH2 region (green) with actin using the FlexPepDock webserver. The actin surface was coloured by electrostatic charge (−3 (red) to +3 kT.e^−1^ (blue)) using the APBS plugin^56^ for PyMOL (The PyMOL Molecular Graphics System, Version 1.8.2 Schrödinger, LLC). Bottom right: The lowest energy docked Tarp:actin complex, with labeled residues, compared to the WAVE2 (pdb code: 2A40) structure (cyan) with a red transparent actin surface.

Finally, NMR was also used to investigate the Tarp:actin interaction for residue-specific perturbations. Two samples were prepared, with approximate actin:Tarp_726–825_ ratios of 0.8:1 and 2:1, keeping actin concentrations low due to polymerization concerns. No additional or significantly shifted peaks were observed in the Tarp_726–825_ [^1^H, ^15^N]-HSQC spectra in the presence of actin, likely due to the large molecular weight of the actin:Tarp_726–825_ complex leading to extreme broadening of Tarp_726–825_ peaks in the bound state (Supplementary Fig. S7). This precluded a direct interrogation of secondary structure for the bound state using NMR. Nonetheless, multiple amide peaks in the [^1^H,^15^N]-HSQC spectrum were shown to have minor chemical shift differences in the presence of actin (Fig. 5, top left). Intensities were normalized to account for experimental differences between the samples. Comparison to the control sample revealed that a large number of peaks had reduced intensity relative to the other peaks in the [^1^H,^15^N]-HSQC spectrum (Supplementary Fig. S7 and Fig. 5, bottom left). Although the normalization process limits the quantitative analysis of Tarp_726-825_ bound to actin, it allows us to map the residues more affected by the presence of actin. These affected residues correspond to a single patch of the Tarp construct, that begins at the proposed α-helix and extends ~10 residues further, although peak broadening is still observed further downstream.

### Modeling the Tarp:G-actin interaction

The combination of sequence similarity, observation of α-helical propensity within the Tarp actin-binding region, and the perturbation of residues C-terminal to the proposed WH2 α-helix, suggest that Tarp could bind actin in a similar manner to other WH2 domain-containing proteins. Although molecular modeling of disordered proteins has its difficulties, as traditional rigid-body *in silico* docking methods cannot sufficiently account for the more extensive protein dynamics, the FlexPepDock webserver^26^ provides a freely available docking methodology whereby only one molecule is portrayed as rigid and an approximate peptide structure of up to 30 residues can be docked under high-resolution low-energy refinement whilst incorporating full peptide backbone flexibility. Built upon the Rosetta framework, FlexPepDock was able to model near native binding conformations when the starting conformation of a peptide ligand was up to 5.5 Å RMSD away from that of the native fold and fully extended during testing^26,27^.

Starting models for Tarp docking were prepared for the complete WH2-aligning region (Tarp sequence in Fig. 3B), built as an extended chain which included an ideal α-helix for the proposed helical residues (A748-K758). As a control, we initially tested the methodology with altered versions of the WAVE2 crystal structure PDB file (2A40). When presented with the native helical fold, the methodology was capable of arriving at structures comparable to the crystal structure (data not shown).

The ten best-performing docked models of the Tarp:actin complex suggest a single conformation for the Tarp peptide wherein the 10-residue Tarp α-helix is bound to the actin hydrophobic cleft (Fig. 5, top right), in good agreement with other WH2 domain-containing proteins. Indeed, the helical residue backbone atoms of all Tarp models aligned to within 0.26 Å RMSD of the WAVE2 conformation. In these models, the C-terminal extended region of Tarp makes contacts with actin subdomain 3, as opposed to subdomain 1 for other WH2 domains, such as the WAVE2 WH2 domain (Fig. 5, bottom right). While we currently lack experimental evidence for this conformation, electrostatic (or potentially salt-bridge) interactions between residues R147, K326, K328 and D761, D767, for actin and Tarp, respectively, could stabilize the interaction. Furthermore, the hydrophobic residues V762 and Y764 from Tarp are within 4 Å of a small hydrophobic pocket on the actin surface (comprising residues G308-A310 and I329-A331). When intermolecular contacts and potential interaction energies were analyzed with software designed for predicting binding affinities (Supplementary Table S2), the native WAVE2 crystal conformation and the lowest energy Tarp:actin model both obtained favorable binding energies, with WAVE2’s affinity somewhat higher than Tarp (as measured), showing that the proposed binding mode is consistent with the experimental results.

## Discussion

The experiments presented herein offer new biophysical insights into how chlamydia species manipulate actin monomers within host cells via the Tarp protein. The distribution of Tarp residues perturbed upon actin binding suggests that a linear, sequential region, homologous to other WH2 domains, is alone sufficient to interact with actin. This is consistent with other WH2 domains, for which the conserved mechanism centers upon a helical interaction within the actin hydrophobic cleft (‘+’ barbed-end) whilst a number of extended residues, contiguous to the helix, facilitate additional interactions with residues across the actin surface^28^.

WH2 domains are often described as predominantly disordered with a propensity to fold upon binding, being cited as an example of a functional disordered domain^29–31^. The Tarp_726–825_ construct, containing the actin-binding region, adds to this consensus, as it shows all the hallmark characteristics of an intrinsically disordered protein: low hydrophobicity, extremely poor NMR spectral dispersion for amide hydrogens, higher apparent molecular mass (by gel filtration chromatography and SDS-PAGE) than expected, and characteristic circular dichroism spectra and temperature-dependent behavior, and is computationally predicted to be disordered.

One of the main functional roles of IDPs is as a protein ‘hub’ in eukaryotes^32^, bringing together multiple binding partners in cell signaling cascades and regulatory processes^33^. The presence of a large number of interaction sites within a single disordered polypeptide chain can also allow for coordinated communication across multiple signaling pathways, acting as efficient multi-function scaffolds^34,35^. Initial disorder predictions for many full length Tarp isoforms indicate ~60–70% disorder. This is consistent with Tarp being a “hub” with multiple mechanisms for actin remodeling: interacting directly with G-actin^7^ or F-actin^5^, or indirectly with vinculin^17^, FAK^16^, or with guanine exchange factors leading to Arp2/3 activation^14,15^. Tarp phosphorylation also leads to interaction with the SHC1 signaling initiator, which modulates native apoptosis and promotes post-infection survival^36^. Additionally, the ability of Tarp to homo-multimerise through its proline-rich domain could lead to further inter-hub networking that may also contribute to infection.

Our results from NMR and SRCD suggest that the 12-residue WH2-homologous patch of Tarp (~A748-H759) samples a partial helical conformation in the free state which can be computationally docked to actin with favorable energies, in a WH2-like fashion, when fully formed. As WH2 domains are intrinsically disordered in the absence of actin^37^, and partially formed helices are observed to different extents in different IDPs^20^, this picture is consistent with the WH2 region of Tarp being disordered, with a limited amount of pre-formed helix, which fully folds upon binding to actin.

When the proposed Tarp actin-binding region is aligned to other documented WH2 domains (Fig. 3B), the Tarp helix shows greatest sequence similarity to the tightest actin-binding WH2 domain, of WAVE2^12^ (44.4% identity). Chereau *et al*. describe the significance of two arginine residues immediately after the helix, in the LRRV motif of WAVE2 (LKKV consensus motif) which are able to form salt bridges to actin residues D24 and D25, in subdomain 1^12^. The LRRV motif is not present in Tarp. Instead, Tarp contains a single negative charge, and a number of hydrophobic residues (LDVVY). These amino acids could be involved in additional hydrophobic interactions, which may help to explain the similar binding affinity.

We have built a computational model for the Tarp:actin interaction based on information derived from our experimental results. As in other homologous WH2 helices, Tarp interacts with the actin subdomain 1–3 hydrophobic cleft, and with residues across the actin surface. However, the position for the C-terminus of Tarp’s actin binding region in our model is different from the equivalent region of WAVE2: Tarp is on the surface of actin subdomain 3, while WAVE2 lies across actin subdomain 1. This location of the C-terminus is not observed in any of the WH2:actin crystal structures currently available, and its significance is unclear. Nonetheless, as the amino acid composition for this region is markedly different, the potential for the Tarp C-terminal region to occupy this alternate location, stabilized by hydrophobic interactions, should not be overlooked. Indeed, an analysis of the binding interfaces in the two structures includes 32 apolar interfacial contacts in our Tarp:actin model, as opposed to 21 in the Wave2:actin crystal structure (Supplementary Table S2). These additional hydrophobic interactions may account for Tarp’s strong affinity to actin (relative to other WH2 domains) in the absence of the electrostatic interactions driven by the lysine side chains. Importantly, this novel subdomain 3 position for the docked C-terminal region of Tarp’s WH2 would not imply any perturbation or steric blocking occurring at or around the nucleotide-binding pocket. Alternatively, the identified positioning of the C-terminus could reflect just one possible position within a “fuzzy” polymorphic bound state, as previously described for disordered WH2 domain-containing proteins^37^. The apparent “fuzziness” of the WH2:actin interaction has been proposed as an underlying mechanism which determines different actin-related functions, such as modulating actin nucleation, polymerization or filament destabilization, in different proteins, depending on the dynamics of the interactions between the WH2 C-terminus and actin subdomains 1 and 2^37^. More recently, it has been suggested that WH2 domains, although essential, play only an auxiliary role in nucleating and elongating actin filaments, by delivering actin monomers to other interacting proteins^28^. Given Tarp’s role as an actin signaling hub protein, its G-actin binding domain is well placed to synergize with recruited host cell actin regulating proteins in order to maximize actin nucleation.

## Methods

### Tarp expression and purification

The GST-Tarp_726–825_ construct from *Chlamydia trachomatis*, previously shown to contain the minimal actin binding fragment^13^, was transformed into competent *Escherichia coli* BL21 (DE3*) cells. Single colonies were used to inoculate LB medium (for the production of unlabelled protein) or MEM (for isotopic labelling). Expression was induced with 0.8 mM IPTG (isopropyl β-D-1-thiogalactopyranoside) when the OD_600_ reached 0.6–0.8. Cultures were grown for a further 3-4 hours and then harvested by centrifugation. Isotopically enriched samples (^15^N or ^15^N/^13^C) were produced in M9 minimal media supplemented with micronutrients and MEM vitamin solution (Sigma), with ^15^NH_4_Cl or ^15^NH_4_Cl and 0.2% ^13^C glucose replacing NH_4_Cl and glucose in the original recipe. Expression was performed overnight at 30 °C. Protein purification was performed using SuperGlu GST-affinity (Generon, UK), according to the manufacturer’s protocol, but using phosphate buffered saline (PBS), pH 7.3 in the resuspension and wash steps. Fractions containing pure GST-Tarp were dialysed exhaustively against PreScission^TM^ cleavage buffer, and digested with PreScission^TM^ protease (GE Healthcare) (2 U.mg^−1^) overnight. Tarp_726–825_ was purified from the GST tag using a 75 cm S75 Superdex (GE Healthcare) chromatography column. Silver stained SDS-PAGE was used to visualise Tarp_726–825_ after digestion as the fragment is not well stained by Coomassie Blue. Tarp_726–825_ samples were transferred to G-actin buffer (0.2 mM CaCl_2_, 2 mM Tris-HCl, pH 7.5, 0.2 mM Na_2_ATP). Na_2_ATP was omitted from NMR and SRCD experiments in the absence of actin.

### G-actin purification

All actin purification steps were derived from those outlined by Pardee and Spudich^38^. The muscle tissue (~240 g) of two freshly sacrificed chickens was used in the initial preparation of “actin-acetone powder” (final yield ~21 g) and stored at −20 °C until use. Samples of G-actin for the biophysical characterizations were purified in G-actin buffer (0.2 mM Na_2_ ATP, 0.2 mM CaCl_2_, 2 mM Tris-HCl, pH 7.5). Actin samples that had been frozen were submitted to a cycle of polymerization and depolymerization to remove any inactive actin.

### NMR sample preparation

Tarp_726–825_ NMR samples were made to a total volume of 500 μL and contained final concentrations of ~1 mM protein in addition to 10% D_2_O, 0.03% w/v NaN_3_ and 200 μM DSS for an internal chemical shift reference. All samples were spin filtered prior to use (0.22 μm, Corning Inc., USA).

### NMR spectroscopy

All NMR spectra were acquired at 298 K either at 800 MHz (Bruker Avance III spectrometer equipped with a triple resonance indirect detect TXI probe with z-gradients), 500 MHz (Bruker Avance I spectrometer with a triple resonance indirect detect TXI probe with z-gradients, or 600 MHz (Varian Inova spectrometer with a 5 mm triple resonance z-gradient probe). The Tarp backbone was assigned through application of the standard “backbone-walk” methodology with the acquisition of CBCA(CO)NH^39^, HNCACB^40^, HNN^41^, and [^1^H-^15^N]-TOCSY-HSQC^42^ spectra. All assignable Tarp chemical shifts have been deposited in the BMRB; accession code 27263. The DSS standard within each sample was used to reference the directly detected ^1^H dimension; all indirect dimensions were referenced according to the ratio of their heteronuclear gyromagnetic ratios and specific nuclear observation frequencies as described by Wishart *et al*.^43^.

### NMR relaxation experiments

All NMR relaxation experiments were acquired at 500 and 800 MHz as a series of interleaved experiments. T_1_ relaxation delays were set to 20, 100, 200, 500, 750, 1000, 2000 and 4000 ms, with duplicates at 20, 500 and 1000 ms. T_2_ relaxation experiments were acquired using a CPMG-based pulse sequence, with a CPMG frequency of 555 Hz. Relaxation delays were set to 18, 54, 90, 144, 180, 216, 270 and 324 ms at 500 MHz, with duplicates at 18, 90 and 180 ms, and to 17, 51, 85, 136, 170, 204, 255 and 306 ms at 800 MHz, with duplicates at 17, 85 and 170 ms. For heteronuclear NOE measurements, interleaved spectra were acquired with and without proton saturation during the recycle delay. Duplicates were used for determination of peak height uncertainties. Recycle delays for all experiments were 7.5 s. Relaxation parameters were fitted using CcpNmr Analysis^44^. Reduced spectral density mapping was carried out manually in Microsoft Excel using equations described by Farrow *et al*.^23^.

### Tarp:actin NMR spectroscopy

Tarp_726–825_:actin NMR samples were prepared by mixing pure samples of Tarp_726–825_ and G-actin in G-actin buffer to the desired ratios. Stock G-actin concentrations were kept below 0.1 mM to prevent polymerisation, leading to sample concentrations of 78 μM actin, 93 μM Tarp_726–825_ (~0.8:1 ratio), and 73 μM actin, 34 μM Tarp_726–825_ (~2:1 ratio). A control Tarp_726-825_ sample was prepared at 93 μM by adding G-actin buffer instead of G-actin. [^1^H-^15^N]-HSQC spectra were acquired at 800 MHz. Chemical shift perturbations (∆δ) were calculated as described by Williamson^45^ using Euclidean weighting correction factors of 0.2 (glycine) and 0.14 (other amino acids). Intensities were normalized in each spectrum using the average intensity of peaks corresponding to residues remaining from the fusion tag. Relative peak intensities were calculated as the ratio between normalized intensities.

### Isothermal titration calorimetry

Isothermal titration calorimetry (ITC) was carried out using a MicroCal 200 calorimeter (GE Healthcare). Prior to experimentation, both Tarp and actin samples were separately exhaustively dialysed against the same G-actin buffer. Ligand binding experiments were carried out at 25 °C with a series of 19 injections, 2 μL each, of 175 μM Tarp (total syringe volume, 40 μL) into a 200 μL chamber of 10 μM G-actin with 1000 rpm stirring. Injections were made over 4 s, 120 s apart, with an initial injection point carried out (volume: 0.4 μL, duration: 0.8 s). Three control experiments were also carried out to identify the enthalpies associated with injection (buffer:buffer), actin dilution (buffer:actin) and Tarp dilution (Tarp:buffer). Only the latter proved significant and the average value of 24 ncal.s^−1^ was subsequently subtracted from each data point prior to analysis. All analyses were carried out within the Origin software package using the integrated MicroCal ITC plugin. Values are the average and standard deviation of four independent experiments.

### Synchrotron radiation circular dichroism spectroscopy

Synchrotron radiation circular dichroism (SRCD) spectra were acquired on beamline CD1 at the Institute for Storage Ring Facilities, University of Aarhus, Denmark, using the same samples that had been prepared for ITC experimentation (Tarp, actin and a 3.6:1 molar ratio of Tarp:actin mixture resulting from completed ITC titration). Measurements were made between 280 nm and 175 nm (1 nm step size) in 0.005 cm pathlength quartz Suprasil cells (Hellma, UK), with an averaging time of 2 s. Variable temperature SRCD was carried out from 20 °C to 90 °C in 5 °C increments, with each step pre-equilibrated for 3 minutes prior to the measurement being made. Three scans acquired at each temperature were averaged, and the average of three baseline scans of the relevant buffer was subtracted. Spectra were calibrated against camphorsulphonic acid^46^ and all processing was carried out with CDTool software^47^. The DichroWeb analysis server was used for all secondary structure determination^48^. Values from the CONTINLL^49,50^ SELCON and CDDSTR^51^ algorithms (using reference dataset 6, which includes 5 denatured proteins)^51^ were averaged and the ± reflect one standard deviation in the values calculated by the 3 methods. The spectra have been deposited in the Protein Circular Dichroism Data Bank^52^ as PCDDB codes: CD0006121000 to CD0006123000 for Tarp-actin binding, and CD0006124000 to CD0006124014 for Tarp thermal melt spectra.

### Docking simulations

*In silico* docking simulations to monomeric G-actin were carried out using the FlexPepDock web server^26^ (accessible: http://flexpepdock.furmanlab.cs.huji.ac.il/). Input files consisted of single PDB-formatted coordinate files containing the G-actin monomer (PDBID 2A40) and relevant peptides. The input model peptide for Tarp consisted of residues 746–767, with an ideal alpha helical backbone geometry (φ/ψ angles of –57 and –47, respectively) enforced for residues 748–758 using MODELLER^53^. The remaining residues were left in an extended conformation. The resulting Tarp peptide was then aligned with WAVE2 and, for additional freedom, translated out of the actin hydrophobic cleft by 5 Å at 90° to the plane of the a-helical axis. All other molecular chains were then deleted. The FlexPepDock methodology was also tested with similar simulations of the 22-residue WAVE2 WH2 domain (residues 433–454) also translated out of the actin hydrophobic cleft.

### Data availability

Backbone resonance assignments for Tarp_726–825_ are available from the BMRB, accession code 27263; SRCD spectra are available from the PCDDB under codes CD0006121000 to CD0006123000 for Tarp-actin binding, and CD0006124000 to CD0006124014 for Tarp thermal melt spectra; NMR relaxation data and any other datasets generated in this study are available from the corresponding author upon reasonable request.

## Electronic supplementary material

Supplementary information
