# Supplementary material for: The intrinsically disordered Tarp protein from chlamydia binds actin with a partially preformed helix
Source: Sci Rep. 2018 Jan 31;8:1960. doi: 10.1038/s41598-018-20290-8 (PMC5792643; doi:10.1038/s41598-018-20290-8)
Supplement: Supplementary file 1 — Supplementary information [file 41598_2018_20290_MOESM1_ESM.pdf]

Supplementary Information

The intrinsically disordered Tarp protein from chlamydia binds actin with a partially preformed helix

James Tolchard[1], Samuel J. Walpole[1], Andrew J. Miles[2], Robin Maytum[3], Lawrence A. Eaglen[1], Ted Hackstadt[4], B. A. Wallace[2], Tharin M. A. Blumenschein[1]

[1]Center for Molecular and Structural Biology, School of Chemistry, University of East Anglia, Norwich Research Park, Norwich, NR4 7TJ, UK. [2]Institute of Structural and Molecular Biology, Birkbeck College, University of London, London, WC1E 7HX, UK. [3]School of Life Sciences, University of Bedfordshire, Park Square, Luton, LU1 3JU, UK. [4]Host-parasite Interactions Section, Laboratory of Intracellular Parasites, NIAID, NIH, Rocky Mountain Laboratories, Hamilton, MT, 59840, USA. Correspondence and requests for materials should be addressed to T.M.A.B. (email: t.blumenschein@uea.ac.uk)

Results

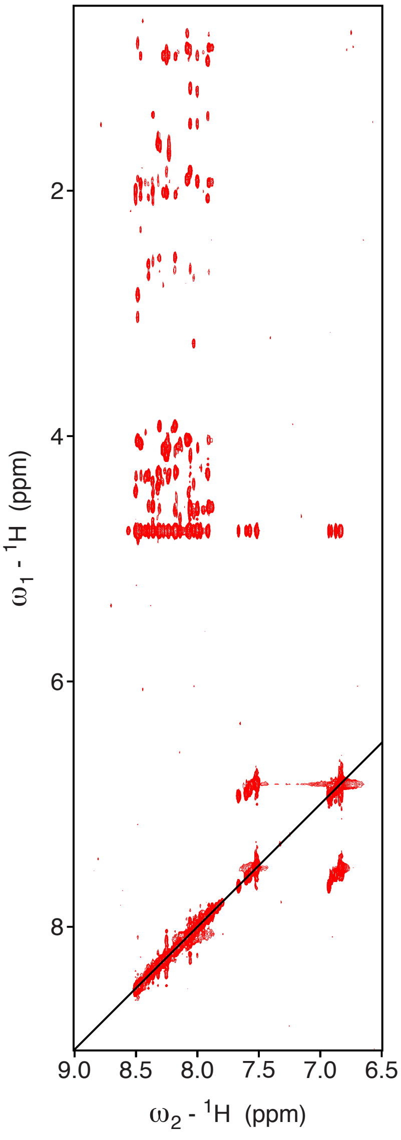

Figure S1. Two-dimensional ^{1}H-^{1}H projection of 3D [^{1}H,^{15}N]-HSQC-NOESY. The spectrum was acquired with a mixing time of 200 ms, at 800 MHz, 298 K. The full ^{15}N spectral width is projected in this image. The few crosspeaks visible correspond to intra-residue correlations. The absence of correlations between amide protons is particularly significant – the only non-diagonal peaks present in the lower half of the spectrum correspond to crosspeaks between two protons in the same glutamine or asparagine side chain.

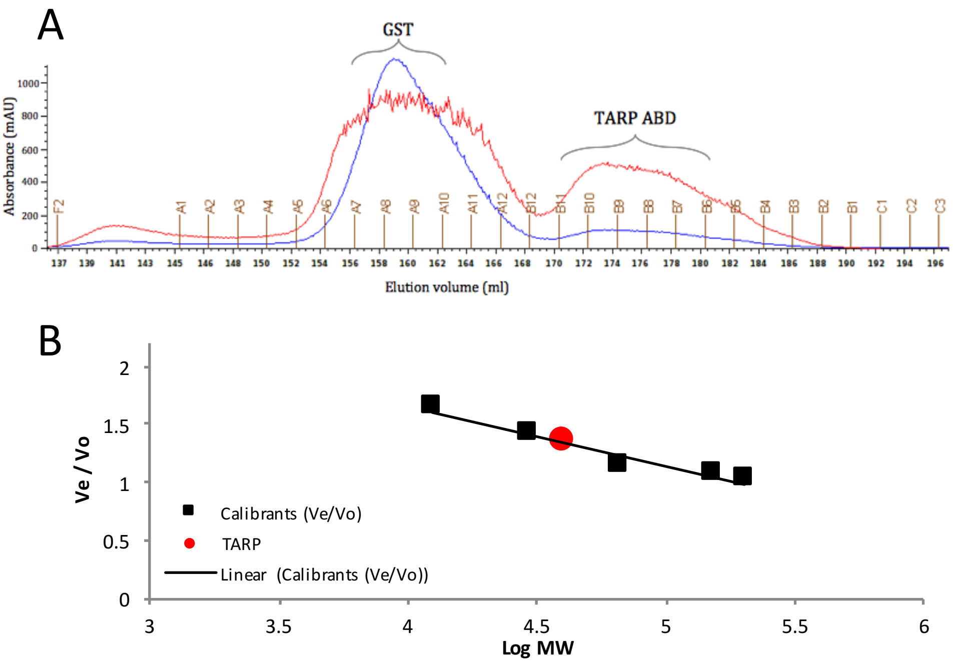

Figure S2. Size exclusion chromatography of $Tarp_{726\text{-}825}$. (**A**) Size exclusion chromatogram of a mixture of GST and $Tarp_{726\text{-}825}$ (after digestion of the GST-$Tarp_{726\text{-}825}$ fusion protein) in a 75 cm S75 Sephadex column, at 280 nm (blue) and 214 nm (red). (**B**) Standard protein calibrants (cytochrome *c*, carbonic anhydrase, bovine serum albumin, alcohol dehydrogenase, and β-amylase; black squares) were used to calibrate the S75 column across a molecular weight range of 12.4 kDa to 200 kDa. The relationship between molecular weight (MW), elution volume (Ve), and void volume (Vo) was used to determine that $Tarp_{726\text{-}825}$ (red circle) elutes from the column with an apparent molecular weight of ~ 40 kDa.

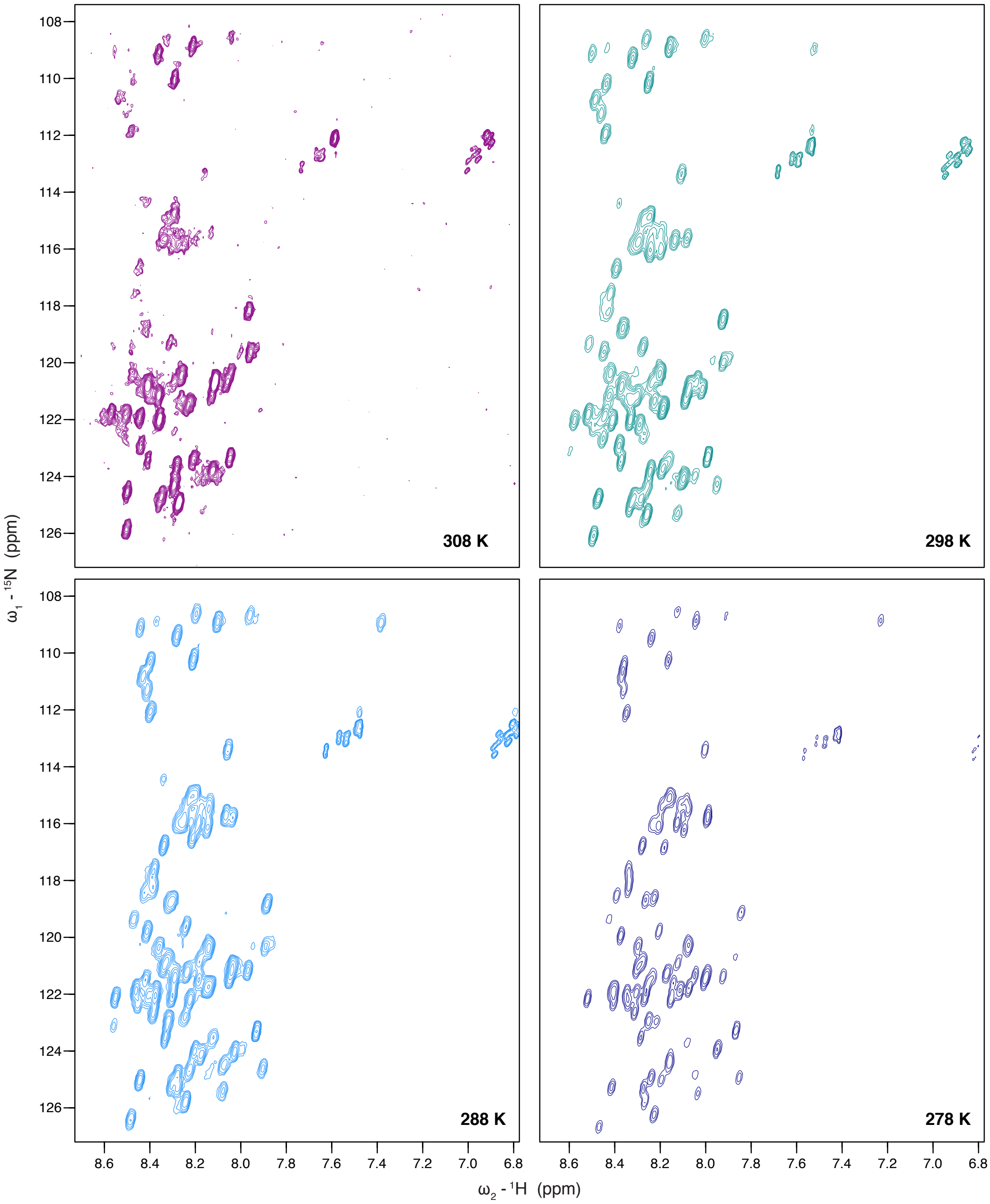

Figure S3. [^{1}H,^{15}N]-HSQC spectra of Tarp$_{726\text{-}825}$ at varying temperatures. Top left, 308 K; top right, 298 K; bottom left, 288 K, bottom right, 278 K. No new peaks potentially corresponding to residues between 808 and 825 were observed.

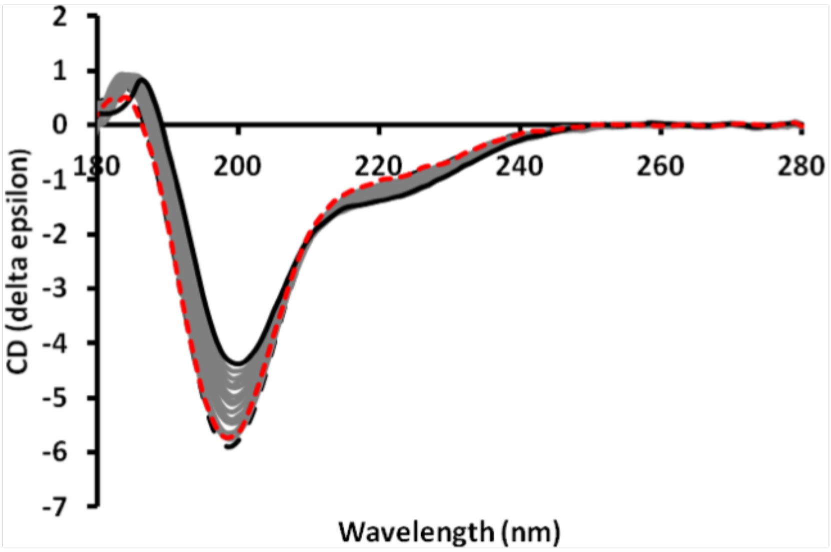

Secondary Structure	20 °C (%)	85 °C (%)	Δ (%)
α-helix	5 ± 2	7 ± 2	2
β-strand	14 ± 2	19 ± 2	5
Disordered	68 ± 2	58 ± 6	-10

Figure S4. Thermal melt synchrotron radiation circular dichroism (SRCD) spectroscopy of Tarp$_{726\text{-}825}$. Top: SRCD spectra of Tarp$_{726\text{-}825}$ measured at 20 °C (dashed black line), and in successively increasing 5 °C increments (solid grey lines) up to 85 °C (solid black line), and a final spectrum after return to 20 °C (dashed red line). **Bottom:** The calculated secondary structure content of Tarp at 20 and 85 °C. Values are the average plus or minus standard deviation from three algorithms (as described in Methods).

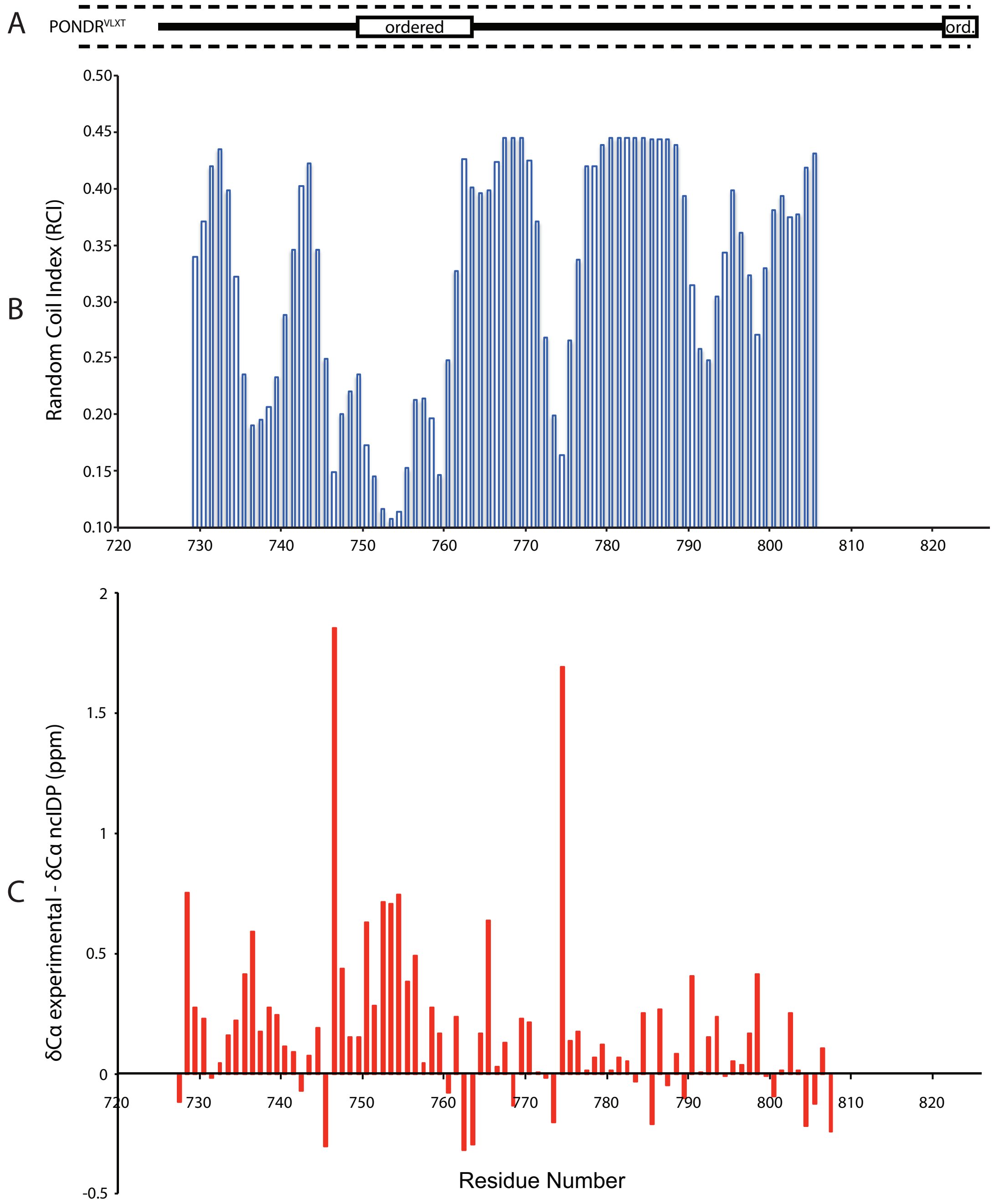

Figure S5. Order and secondary structure. (**A**) PONDR-VLXT[1,2] *in silico* disorder prediction (default parameters) for the $Tarp_{726\text{-}825}$ amino acid sequence. (**B**) The random coil index value as calculated by the RCI webserver[3] using the $Tarp_{726\text{-}825}$ backbone chemical shifts with default parameters and Wishart random coil values[4]. (**C**) Secondary neighbor-corrected Cα chemical shifts, calculated using Tamiola *et al*. random coil values for intrinsically disordered proteins[5].

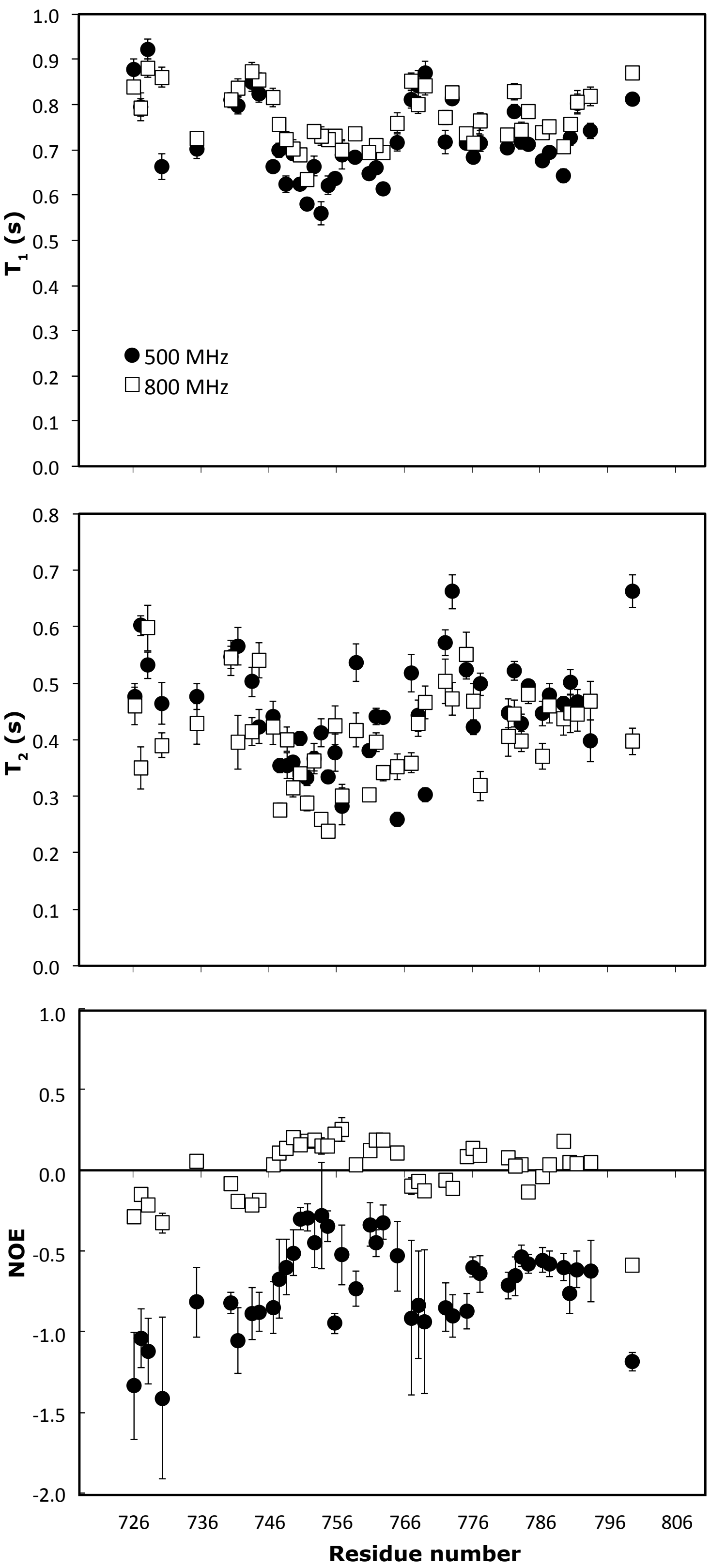

Figure S6. ^{15}N relaxation parameters. T_1, T_2 and heteronuclear NOE were measured for $Tarp_{726\text{-}825}$ at 500 (black circles) and 800 MHz (white squares), at 298 K, in G-actin buffer, pH 7.5. Error bars correspond to the fitting errors for each parameter.

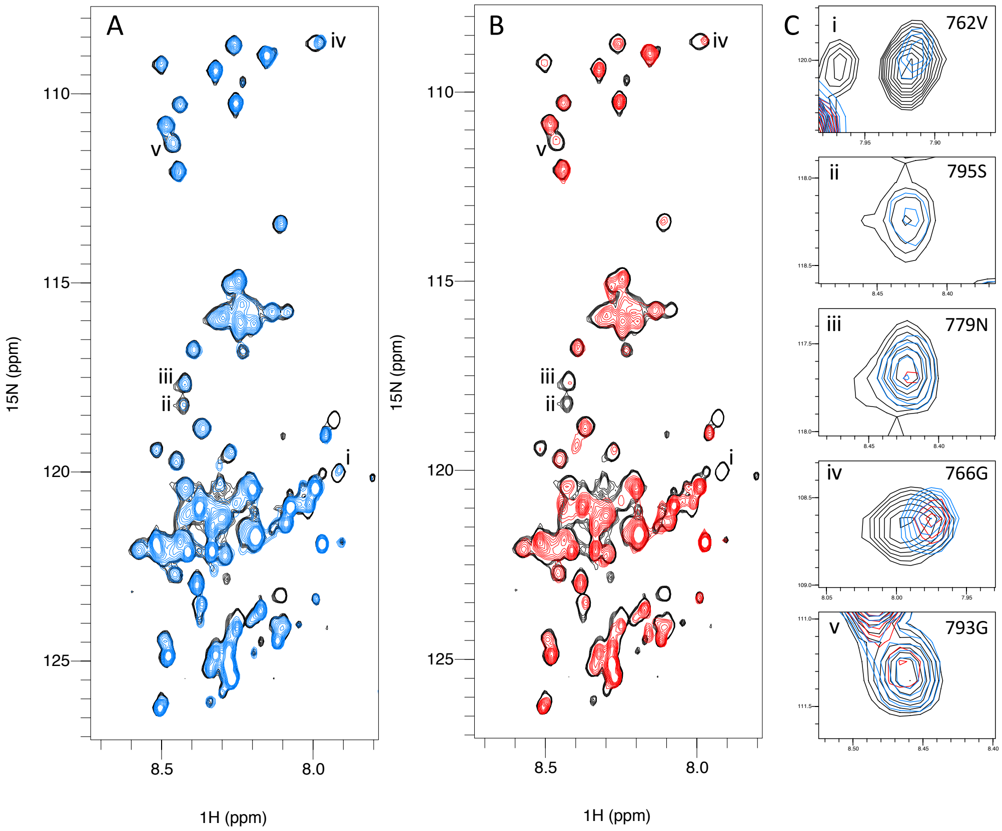

Figure S7. Binding of Tarp$_{726\text{-}825}$ to actin observed by NMR spectroscopy. (**A**) and (**B**) The central region of [^{1}H-^{15}N]-HSQC Tarp$_{726\text{-}825}$ spectra in the absence of actin (black), in the presence of 0.8:1 actin:Tarp ratio (blue) and of 2:1 actin:Tarp ratio (red). A few examples of peaks with significant decreases in intensity between 0.8:1 and 2:1 actin:Tarp ratios are highlighted with roman numerals. (**C**) Enlarged view of the peaks highlighted in (**A**) and (**B**). Spectra were scaled according to the peak intensity of residues remaining from the fusion tag.

**ITC fitted parameters**	
n	1.00 ± 0.06
K_d	102 ± 33 nM
ΔH	–16.10 ± 0.71 kcal mol^{-1}
–TΔS	6.56 ± 0.91 kcal mol^{-1}
ΔG	–9.54 ± 0.21 kcal mol^{-1}

Table S1. Tarp:actin binding parameters. Parameters are shown as average and standard deviation of four independent experiments.

	Tarp:actin	WAVE2:actin
K_d (experimental)	102 nM	52 nM[6]
K_d ^{P}	3.4 µM	440 nM
ΔG (experimental)	-9.54 kcal.mol^{-1}	unknown
ΔG ^{P}	-7.5 kcal.mol^{-1}	-8.7 kcal.mol^{-1}
**FOLDX[7] Interaction energy**	-12.16 kcal.mol^{-1}	-13.98 kcal.mol^{-1}
**Interfacial contacts (ICs)** ^{P}		
ICs charged-charged	2	6
ICs charged-polar	3	2
ICs charged-apolar	12	16
ICs polar-polar	1	0
ICs polar-apolar	10	10
ICs apolar-apolar	32	21
**Non Interacting Surface (NIS)** ^{P}		
%NIS charged	29.68	30.47
%NIS apolar	42.05	40.14

Table S2. Modeled and measured thermodynamic parameters for the Tarp and WAVE2, G-actin interaction. Parameters denoted ^{P} were calculated using the PRODIGY webserver[8].

References

1. Romero, P., Obradovic, Z. & Dunker, A. K. Sequence data analysis for long disordered regions prediction in the calcineurin family. *Genome Inf. Ser Work. Genome Inf.* **8,** 110–124 (1997).
2. Li, X., Romero, P., Rani, M., Dunker, A. K. & Obradovic, Z. Predicting protein disorder for N-, C-, and internal regions. *Genome Inf. Ser Work. Genome Inf.* **10,** 30–40 (1999).
3. Berjanskii, M. V. & Wishart, D. S. A simple method to predict protein flexibility using secondary chemical shifts. *J. Am. Chem. Soc.* **127,** 14970–14971 (2005).
4. Wishart, D. S. & Sykes, B. D. Chemical shifts as a tool for structure determination. *Methods Enzymol.* **239,** 363–392 (1994).
5. Tamiola, K., Acar, B. & Mulder, F. A. A. Sequence-specific random coil chemical shifts of intrinsically disordered proteins. *J. Am. Chem. Soc.* **132,** 18000–3 (2010).
6. Chereau, D. *et al.* Actin-bound structures of Wiskott-Aldrich syndrome protein (WASP)-homology domain 2 and the implications for filament assembly. *Proc. Natl. Acad. Sci. U. S. A.* **102,** 16644–16649 (2005).
7. Schymkowitz, J. *et al.* The FoldX web server: an online force field. *Nucleic Acids Res.* **33,** W382-388 (2005).
8. Xue, L. C., Rodrigues, J. P., Kastritis, P. L., Bonvin, A. M. & Vangone, A. PRODIGY: A web server for predicting the binding affinity of protein-protein complexes. *Bioinformatics* **32,** 3676–3678 (2016).
